# Ultrastructural and Molecular Analysis of Ribose-Induced Glycated Reconstructed Human Skin

**DOI:** 10.3390/ijms19113521

**Published:** 2018-11-08

**Authors:** Roberta Balansin Rigon, Sabine Kaessmeyer, Christopher Wolff, Christian Hausmann, Nan Zhang, Michaela Sochorová, Andrej Kováčik, Rainer Haag, Kateřina Vávrová, Martina Ulrich, Monika Schäfer-Korting, Christian Zoschke

**Affiliations:** 1 Institute of Pharmacy (Pharmacology & Toxicology), Freie Universität Berlin, Königin-Luise-Str. 2+4, 14195 Berlin, Germany; roberta_rigon@yahoo.com.br (R.B.R.); christopher.wolff@fu-berlin.de (C.W.); christian.hausmann@fu-berlin.de (C.H.); nan.zhang@fu-berlin.de (N.Z.); Monika.Schaefer-Korting@fu-berlin.de (M.S.-K.); 2 Institute of Veterinary Anatomy, Department of Veterinary Medicine, Freie Universität Berlin, Koserstr. 20, 14195 Berlin, Germany; sabine.kaessmeyer@fu-berlin.de; 3 Faculty of Pharmacy, Charles University, Akademika Heyrovského 1203, 50005 Hradec Králové, Czech Republic; sochormi@faf.cuni.cz (M.S.); kovacika@faf.cuni.cz (A.K.); katerina.vavrova@faf.cuni.cz (K.V.); 4 Institute of Chemistry and Biochemistry, Freie Universität Berlin, Takustr. 3, 14195 Berlin, Germany; Germany; haag@chemie.fu-berlin.de; 5 Collegium Medicum Berlin, Luisenstr. 54, 10117 Berlin, Germany; ulrich@dermatologie-am-regierungsviertel.de

**Keywords:** advanced glycated end products, aging, diabetes, electron microscopy, nanomedicine, ribose, reconstructed human skin, skin absorption

## Abstract

Aging depicts one of the major challenges in pharmacology owing to its complexity and heterogeneity. Thereby, advanced glycated end-products modify extracellular matrix proteins, but the consequences on the skin barrier function remain heavily understudied. Herein, we utilized transmission electron microscopy for the ultrastructural analysis of ribose-induced glycated reconstructed human skin (RHS). Molecular and functional insights substantiated the ultrastructural characterization and proved the relevance of glycated RHS beyond skin aging. In particular, electron microscopy mapped the accumulation and altered spatial orientation of fibrils and filaments in the dermal compartment of glycated RHS. Moreover, the epidermal basement membrane appeared thicker in glycated than in non-glycated RHS, but electron microscopy identified longitudinal clusters of the finest collagen fibrils instead of real thickening. The *stratum granulosum* contained more cell layers, the morphology of keratohyalin granules decidedly differed, and the *stratum corneum* lipid order increased in ribose-induced glycated RHS, while the skin barrier function was almost not affected. In conclusion, dermal advanced glycated end-products markedly changed the epidermal morphology, underlining the importance of matrix–cell interactions. The phenotype of ribose-induced glycated RHS emulated aged skin in the dermis, while the two to three times increased thickness of the *stratum granulosum* resembled poorer cornification.

## 1. Introduction

Age-related changes in human physiology become increasingly relevant, because life expectancy is rising as fast as the number of geriatric and often multimorbid patients. Aging comprises the functional impairment of cells, tissues, and organs, which promotes diseases like diabetes or cancer [1]. However, drug treatment of elderly patients remains challenging, as drug development mainly focuses on tests in juvenile cells and humans. While drug metabolism and elimination are known to change over lifetime, alterations in the pharmacodynamics of drugs depict a significant knowledge gap—not only in dementia [2].

One hallmark of aging is the accumulation of advanced glycated end-products (AGEs), although increased amounts of AGEs are also found in diabetes and psoriasis [3,4]. Being exclusively removed by protein turn over, AGEs accumulate in proteins with long half-lives. Thus, the collagen half-life of 15 years in skin and 117 years in cartilage fosters the accumulation of AGEs [5]. As products of the non-enzymatic reaction of sugars or aldehydes with proteins, AGEs comprise a heterogeneous group of molecules including pentosidine and *N*-carboxymethyl-lysine (CML).

Several nonclinical models of skin aging tried to emulate the long process of aging in vitro, by exposing juvenile reconstructed human skin (RHS) either to ribose, glyoxal, glyceraldehyde, or specific AGEs [6,7,8,9,10]. As the consequences of extracellular matrix (ECM) glycation depend on the stressor in vitro and strongly deviate among the protocols for RHS glycation, we were intrigued in the unknown effects of AGEs in RHS on epithelial proliferation and differentiation.

Herein, we studied the effects of ECM glycation on the ultrastructure of cellular and extracellular parts of RHS by transmission electron microscopy (TEM) and compared the morphology to juvenile, non-glycated RHS. TEM proved already useful to study subcellular interactions during vascular morphogenesis in 3D cocultures between endothelial cells and fibroblasts [11]. Moreover, we investigated the morphology of ribose-induced glycated RHS by reflectance confocal microscopy, being clinically used for the evaluation of skin cancer and aging [12,13]. Next, we analyzed the protein expression as well as the *stratum corneum* lipid prolife and their spatial orientation. Finally, we studied the effect of collagen glycation on the absorption of reference compounds and the efficacy of an investigative new drug delivery system.

## 2. Results

### 2.1. Selection of Glycation Agent

**Figure 1:**
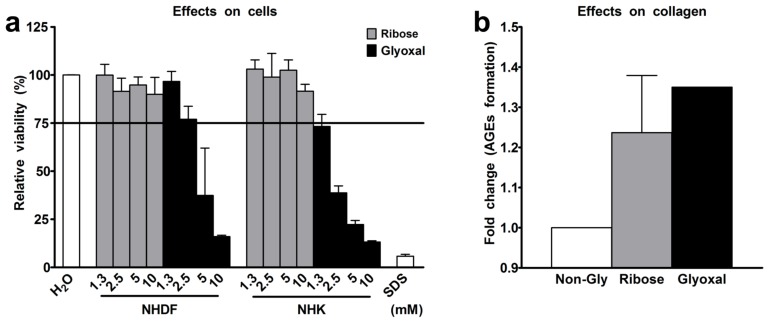
Selection of glycation agent. (**a**) Viability of normal human dermal fibroblasts (NHDF) and normal human keratinocytes (NHK) following exposure to ribose or glyoxal for 48 h. (**b**) Advanced glycation end-products (AGEs) in collagen following the exposure to 10 mM ribose or 10 mM glyoxal. *n* = 3, mean + SD.

We exposed normal human dermal fibroblasts (NHDF) and keratinocytes (NHK) either to ribose or to glyoxal for 48 h. Next, we tested the efficiency of both glycation agents in producing AGEs in collagen following a three-week exposure to either ribose or glyoxal. The viability of both fibroblasts and keratinocytes markedly declined by up to 87% even in trace concentrations of the reagents (Figure 1a). As ribose and glyoxal induced comparable amounts of AGEs in collagen (Figure 1b), we selected the collagen, glycated with ribose, to build glycated RHS. After three weeks of RHS culture, we still detected 1.4-fold increased levels of *N*-carboxymethyl-lysine (CML), a hallmark AGE, in the dermal compartment of glycated RHS. The accumulation of CML was even higher (1.9-fold increase) in the epidermal compartment, although ribose has been removed from the collagen by dialysis prior to RHS building. Accordingly, the expression of the receptor of AGEs (RAGE) was elevated in the entire glycated RHS (Section 2.5).

### 2.2. RHS Morphology

**Figure 2:**
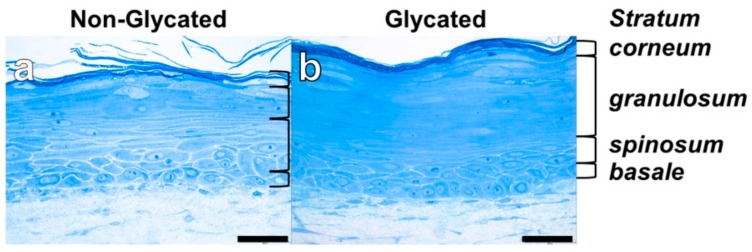
Reconstructed human skin (RHS) morphology. Semi-thin sections of (**a**) non-glycated and (**b**) glycated RHS. Light microscopy, bar = 50 µm.

Both glycated and non-glycated RHS showed a stratified epidermis, but the epidermal compartment of glycated RHS was 1.3 ± 0.18-fold thicker than the epidermis of non-glycated RHS. Moreover, glycated RHS appeared yellowish during the culture period (Figure 2).

### 2.3. Dermal Ultrastructure

**Figure 3:**
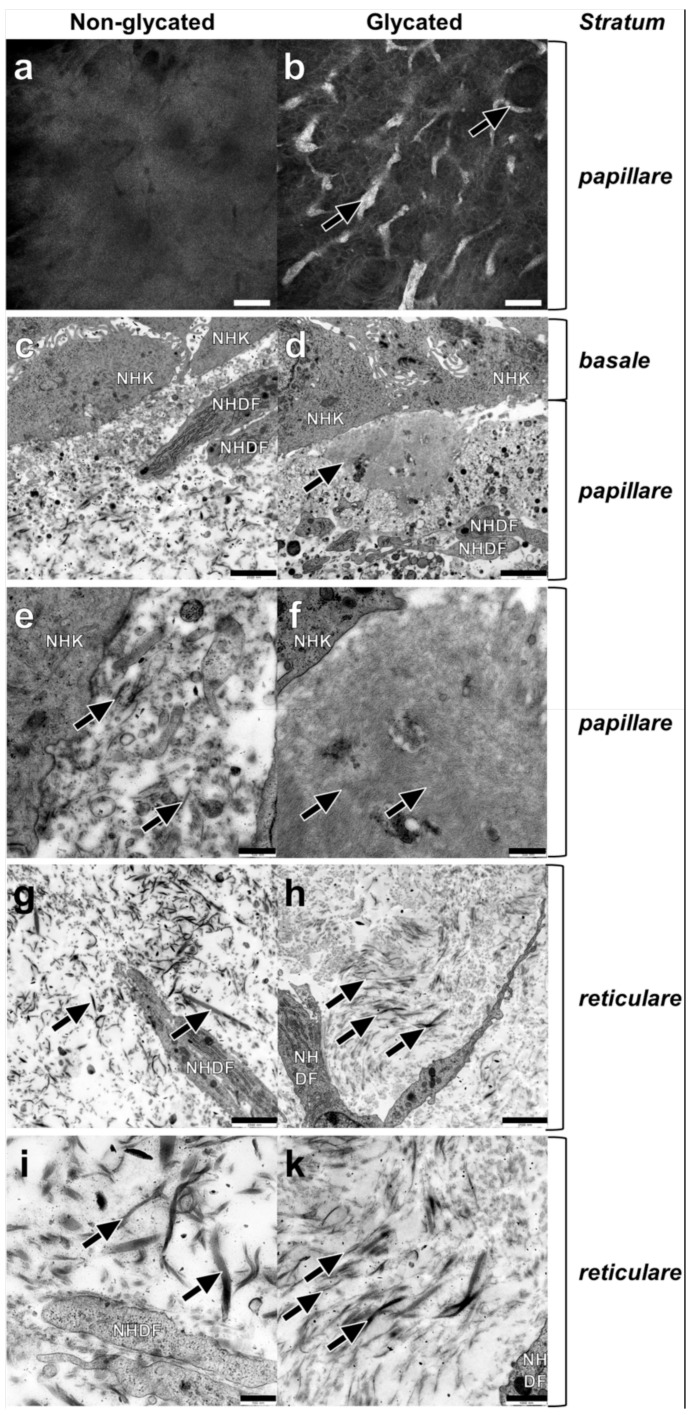
Dermal ultrastructure of (**a**,**c**,**e**,**g**,**i**) non-glycated and (**b**,**d**,**f**,**h**,**k**) glycated RHS**.** (**a**) Homogenous and (**b**) aggregated collagen in the *stratum papillare*. (**c**–**f**) Ultrastructure of the *stratum papillare*. Fibroblasts (NHDF), cell fragments, and vesicles, as well as filaments and fibrils of various sizes bordering the keratinocytes (NHK) of the *stratum basale.* (**c**,**e**) Lower concentration of the structural elements in non-glycated dermal compartment filaments with ((**e**) magnification of (**c**)) fibrils of various sizes (arrows). (**d**,**f**) Local accumulations of thin, densely organized structures in glycated RHS, nearby the *stratum basale* (arrow) ((**f**) magnification of (**d**)), finest cross-linked filaments with a wave-like pattern constitute densely organized structures (arrows). (**g**–**k**) Ultrastructure of *stratum reticulare*. (**g**,**i**) Collagen fibers (arrows) show a disorientated pattern in all directions ((**i**) magnification of (**g**)). Collagen fibers with different sizes in non-glycated RHS (arrows). (**h**,**k**) Collagen fibrils axially orientated to the *stratum basale* ((**k**) magnification of (**h**)) the thinner the fibrils, the more densely they are packed. Densely packed fine fibrils and loosely packed thicker fibrils (arrows). (**a**,**b**) Reflectance confocal microscopy, bar = 100 µm; (**c**,**d**,**g**,**h**) transmission electron microscopy (TEM), bar = 2.5 µm; (**e**,**f**,**i**,**k**) TEM, bar = 1.0 µm.

Next, we analyzed the dermal morphology of glycated RHS and compared it with non-glycated RHS. The dermal compartment was divided into a *stratum papillare,* connected to the *stratum basale* of the epidermal compartment and the deeper *stratum reticulare.* Reflectance confocal microscopy (RCM) proved a homogenous dermal compartment in non-glycated RHS (Figure 3a) and collagen aggregates (white structures in Figure 3b) in the *stratum papillare* of glycated RHS. These findings were corroborated by TEM, where aggregates appeared as an accumulation of finest fibrils in the *stratum papillare* of glycated RHS. Those fibrils either orientated themselves longitudinally to the basal layer (mimicking a punctually developed basement membrane) or were cross-linked into densely organized ellipsoid structures, comprising an area of approximately 8.4 µm × 5.0 µm (Figure 3d). The fine fibrils within these structures were virtually uniformly parallel and appeared in a wave-like pattern (Figure 3f). Furthermore, areas with a locally higher density of collagen fibrils were identified in both *stratum papillare* and *stratum reticulare* of the glycated RHS. Fibrils of about 38 nm diameter appeared densely-packed while fibrils of about 160 nm were loosely packed. It is noteworthy that the collagen fibrils are aligned parallel to the *stratum basale* in the glycated RHS (Figure 3h,k). In contrast, filaments and collagen fibrils aligned randomly in both parallel and perpendicular directions to the *stratum basale* within *stratum reticulare* of non-glycated RHS (Figure 3g,i). Overall, the fibrils and filaments accumulated locally in the glycated RHS, while they were disseminated in the non-glycated RHS. Interestingly, fibroblasts within both RHS exhibited a spread shape and contained a large amount of rough endoplasmic reticulum. Moreover, on average, fibroblast numbers in the *stratum papillare* exceeded those in the *stratum reticulare* about two-fold in both RHS.

### 2.4. Epidermal Ultrastructure

**Figure 4:**
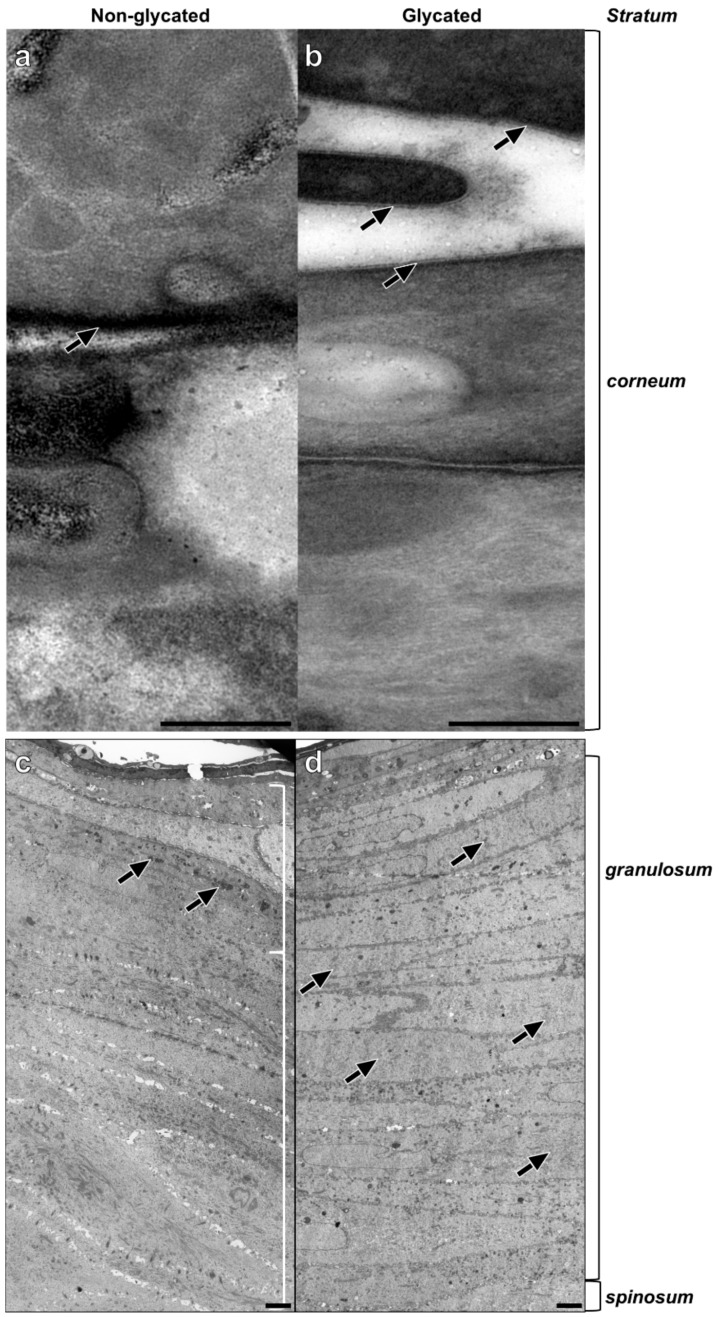
Epidermal ultrastructure of (**a**,**c**) non-glycated and (**b**,**d**) glycated RHS. (**a**) Lipid lamellae within the intercellular space of neighboring corneocytes. (**b**) Fine lines of the intercellular lipids are clearly visible (arrows). (**c**) Packed keratohyalin granule-like structures (arrows) in the upper layers of the viable epidermis, indicating the *stratum granulosum*. (**d**) The *stratum granulosum* dominates the epidermal compartment. Fine electron dense material evenly disseminated within the cytoplasm of the keratinocytes (arrows). Pictures are representative for two batches. (**a**,**b**). TEM, bar = 0.25 µm; (**c**,**d**) TEM, bar = 2.5 µm.

TEM analysis of the epidermal compartments revealed striking differences between glycated and non-glycated RHS, especially in the thickness and number of cell layers of the *stratum granulosum* (Figure 4c,d).

The *stratum spinosum* of non-glycated RHS contained seven layers on average, comprising a decidedly greater proportion of the viable epidermis than in glycated RHS. The *stratum granulosum* of non-glycated RHS constituted on average three layers of tightly packed cells with mitochondria and large, irregularly shaped keratohyalin granules (Figure 4c).

In the glycated RHS, the keratinocytes in the basal layer were columnar and cuboidal shaped. The *stratum spinosum* contained four layers on average, while more than 20 cell layers were designated as *stratum granulosum*, comprising the majority of keratinocytes within the epidermis of glycated RHS. These keratinocytes were increasingly flattened and densely packed. Atypical keratohyalin granules were present as fine, disseminated electron dense material. Although this material slightly differed from the keratohyalin granules in non-glycated RHS, we regarded this material as small keratohyalin granules. Moreover, the number of mitochondria and other cell organelles decreased, especially in the apical parts of the *stratum granulosum* (Figure 4d).

In both constructs, the keratinocytes transformed into flat, tightly packed corneocytes, attaching themselves to each other via corneodesmosomes. *Stratum corneum* of non-glycated RHS consisted of seven layers of corneocytes, which increased to twelve layers in glycated RHS. While a cornified envelope was developed in both types of RHS, the lipids in the glycated models were slightly clearer to visualize and distinguish from the plasma membrane than in non-glycated RHS (Figure 4a,b). This deviating ultrastructure points to different lipid organization in non-glycated and glycated RHS.

### 2.5. Molecular Analysis

**Figure 5:**
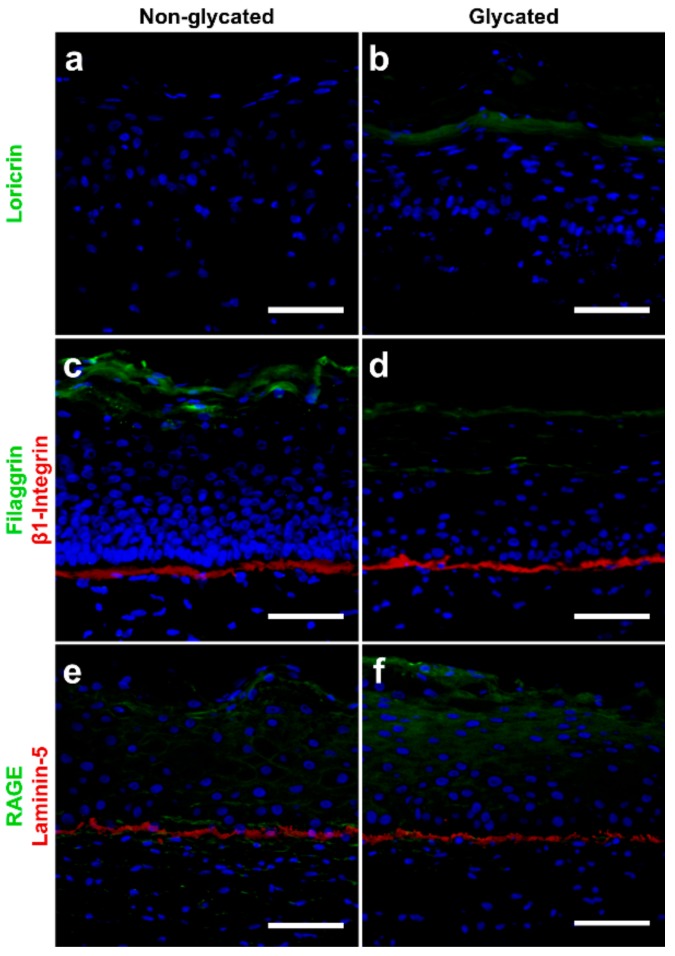
Immunolocalization of proteins in (**a**,**c**,**e**) non-glycated and (**b**,**d**,**f**) glycated RHS. Glycated RHS showed (**a**,**b**) loricrin upregulation, (**c**,**d**) filaggrin downregulation and slight increase in β1-integrin expression, and (**e**,**f**) slight upregulation of both receptor of AGEs (RAGE) and laminin-5 expression compared with non-glycated RHS. Proteins in green or red, cell nuclei (DAPI staining) in blue. Fluorescence microscopy, bar = 100 µm. Pictures are representative for two to four batches.

The expressions of the adhesion molecule β1-integrin and laminin-5 slightly increased within the basal layer of the glycated RHS and faded in the suprabasal epidermal layers (Figure 5). In contrast, filaggrin expression markedly decreased and loricrin expression strongly increased in glycated compared with non-glycated RHS.

**Figure 6:**
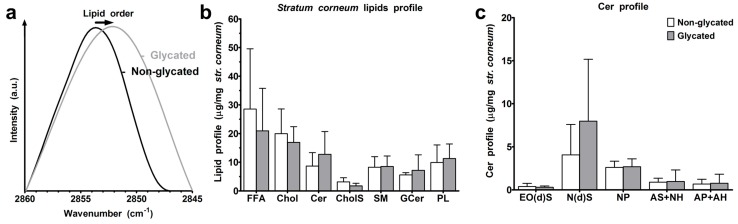
*Stratum corneum* lipids. (**a**) Lipid order. (**b**) *Stratum corneum* lipid profile of non-glycated and glycated RHS for free fatty acids (FFA), cholesterol (Chol), ceramides (Cer), cholesterol sulphate (CholS), sphingomyelin (SM), glucosylceramides (GCer), and phospholipids (PL). (**c**) Ceramide profile, for ceramide nomenclature, see Figure 8; *n* = 3–4, mean + SD.

The arrangement of the intercellular lipids in the *stratum corneum* was probed by Fourier-transform infrared (FT-IR) spectroscopy. The lower wavenumbers of the methylene symmetric stretching vibrations (2851.8 ± 0.7 cm^−1^ in glycated compared with 2852.8 ± 1.0 cm^−1^ in non-glycated RHS, *p* > 0.05) indicated a higher proportion of all-trans chain conformers in the chain [14], and thus an improved lipid chain order in glycated RHS (Figure 6a). High performance thin layer chromatography of *stratum corneum* lipids revealed slight compositional differences. The *stratum corneum* lipids in the non-glycated RHS were composed of 7:5:1 FFA/Chol/Cer, while glycated RHS revealed a molar ratio of 5:3:1 (FFA/Chol/Cer). The absolute amounts of free fatty acids (FFA), cholesterol (Chol), and cholesterol sulphate (CholS) apparently decreased in glycated RHS (*p* ≤ 0.05), while the levels of the levels of ceramide precursors remained unchanged (*p* > 0.05). The respective values read as follows: FFA, 28.5 ± 21.0 and 20.9 ± 14.8 μg/mg *stratum corneum* in non-glycated and glycated RHS; Chol, 19.9 ± 8.6 and 16.9 ± 5.5 μg/mg; and CholS, 3.1 ± 1.4 and 1.8 ± 0.9 μg/mg (*p* = 0.051). The respective amounts of Cer precursors read sphingomyelin (SM) 8.3 ± 3.7 and 8.5 ± 3.7 μg/mg, glucosylceramides (GCer) 5.6 ± 0.8 and 7.2 ± 5.4 μg/mg, and phospholipids (PL) 9.9 ± 6.1 and 11.3 ± 5.1 μg/mg (Figure 6b). The amounts of ceramides (Cer) subclasses, Cer EO(d)S (0.39 ± 0.37 and 0.31 ± 0.15 μg/mg, *p* > 0.05), Cer NP (2.62 ± 0.71 and 2.69 ± 0.91 μg/mg, *p* > 0.05), Cer AS+NH (0.90 ± 0.46 and 0.98 ± 1.34 μg/mg, *p* > 0.05), and Cer AP+AH (0.68 ± 0.56 and 0.78 ± 1.05 μg/mg, *p* > 0.05) did not change. In contrast, the total amounts of Cer (8.7 ± 4.7 and 12.7 ± 7.9 μg/mg, *p* > 0.05) and, in particular, Cer N(d)S (4.06 ± 3.53 and 7.98 ± 7.18 μg/mg, *p* > 0.05) apparently increased in glycated RHS (Figure 6b,c).

### 2.6. Skin Barrier Function

**Figure 7:**
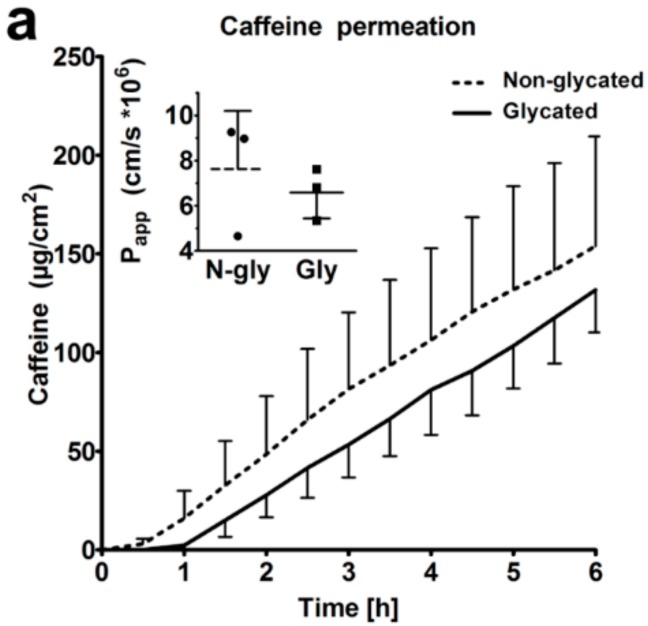

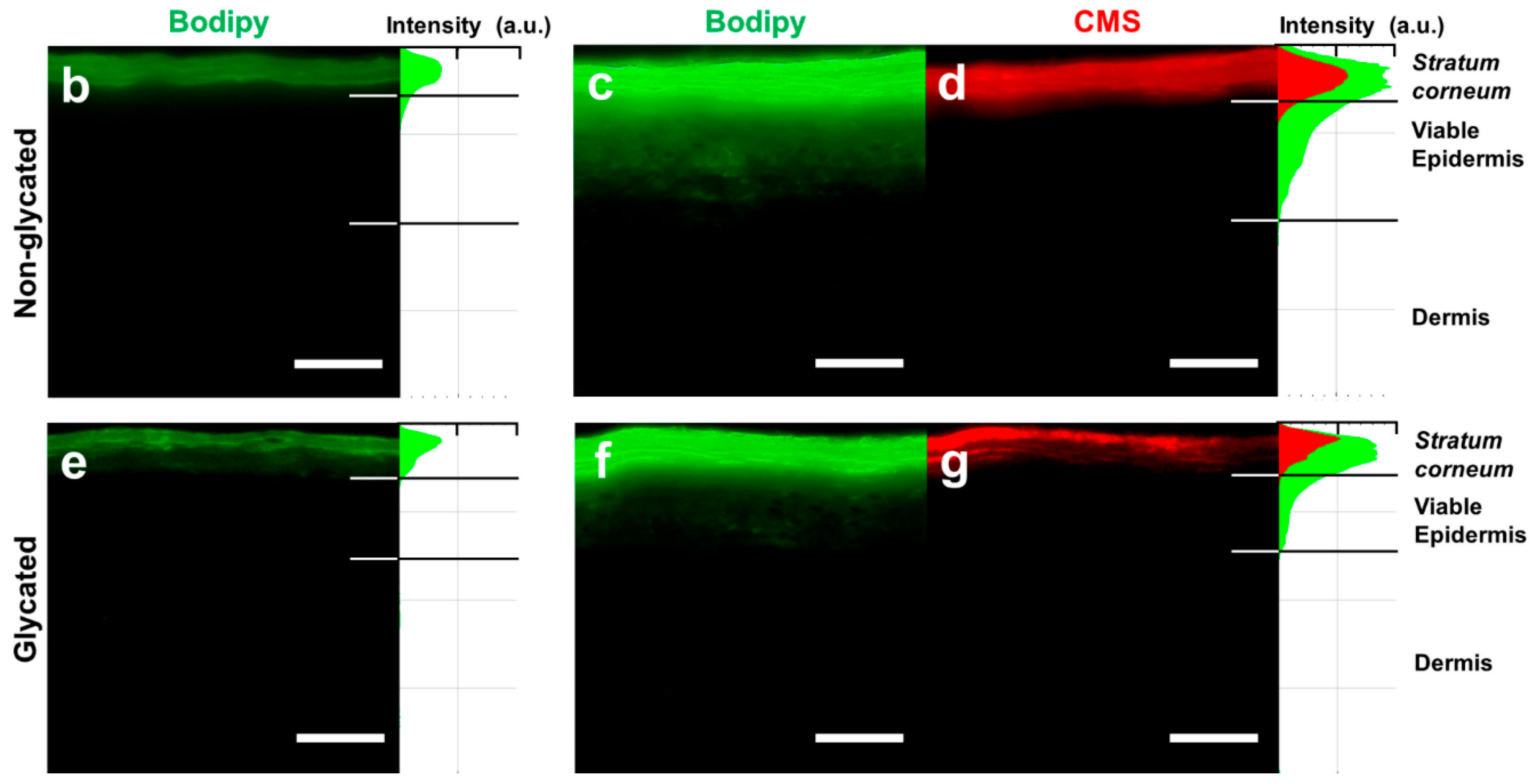
Skin barrier function. (**a**) Caffeine permeation through non-glycated and glycated RHS. The inset shows the respective P_app_ values. (**b**) Penetration of Bodipy cream and (**c**,**d**) Bodipy-core-multishell (CMS) into non-glycated RHS. (**e**) Penetration of Bodipy cream and (**f**,**g**) Bodipy-CMS into glycated RHS. (**c**,**d**,**f**,**g**) CMS labelled with red fluorescent indocarbocyanine. Fluorescence microscopy, bar = 100 µm, *n* = 3, mean ± SD.

Next, we studied the barrier function of glycated RHS. We found comparable caffeine permeation over 6 h (Figure 7a), with apparent permeability (P_app_) values of 7.6 and 6.6 × 10^−6^ cm/s (*p* > 0.05) in non-glycated and glycated RHS, respectively. The lag-time increased from 26 min in non-glycated to 44 min in ribose-induced glycated RHS. However, this trend was not statistical significant.

Finally, we evaluated the delivery of the fluorescent drug surrogate Bodipy, either in base cream or loaded to dendritic core-multishell (CMS) nanotransporters into RHS (Figure 7b–g). Bodipy and CMS nanotransporters penetrated the *stratum corneum* of both glycated and non-glycated RHS. While Bodipy readily penetrated viable epidermal layers in the non-glycated RHS, this uptake appeared slightly reduced in glycated RHS (Figure 7c,f). However, Bodipy uptake was enhanced by CMS nanotransporters compared with cream in both non-glycated and glycated RHS (Figure 7b,c,e,f).

## 3. Discussion

The complex and heterogeneous nature of skin aging challenges the relevant and reliable emulation of ageing hallmarks in vitro. However, RHS proved to be a powerful tool to mimic at least some important features of skin diseases like atopic dermatitis, psoriasis, and non-melanoma skin cancer [15,16,17]. In order to mimic aged skin, the ECM of juvenile RHS was exposed to either ribose glyoxal or specific AGEs. These artificial glycation stimuli are necessary to speed up the slow amino carbonylation by glucose. We selected ribose because of its biocompatibility and its glycation efficiency (Figure 1). In contrast to glyoxal, ribose did not affect the viability of skin cells. Moreover, ribose not only produces structurally similar AGEs [18], but also reacts faster than glucose [19]. Nevertheless, the effects of glycation appear to depend on the glycation agent; with ribose and glyoxal being the most frequently used compounds [8,9,20]. Granted that aged skin is sufficiently perfused in vivo, the oxidative environment should favor glycation by sugars [21].

The increased stiffness of the dermal ECM by collagen cross-linking depicts one hallmark of aged skin [22]. Ribose glycation reliably mimicked this morphology as reflectance confocal microscopy showed collagen aggregates and the ultrastructural analysis revealed the altered alignment and higher amounts of collagen fibers and fibrils in glycated RHS (Figure 3). Moreover, we proved the interdependency between fibril diameter and packing, as well as a hierarchy of fiber accumulation. While densely packed fibrils are smaller in diameter, loosely packed collagen fibrils showed larger diameters (Figure 3). The overall architecture of the dermal compartment in glycated RHS showed more or less axially orientated collagen fibrils. Cross-linking of the fibrils resulted in their dissociation to finer structures. Accumulations of increasingly finer cross-linked fibrils in densely packed structures resembled stiffly plaques within the skin. These changes in ECM also explain the lost dermal flexibility in diabetic patients [3] and are not limited to skin, but apply also to glycated vascular walls or tendons. In accordance, glycation in vivo increases fibril packing density, the range of fibril diameters, and the fusion of fibrils in tendons of diabetes patients [4].

The effects of dermal collagen glycation go beyond the mechanical alterations of dermal ECM. This is particular intriguing, as epidermal changes are induced either by traces of ribose (despite of the extensive dialysis to eliminate the glycation agent) or by AGEs or by altered paracrine signaling between fibroblasts and keratinocytes in glycated RHS. We argue for the latter, because the mechanical separation of glycated ECM and the epidermal compartments did not impair the effects of AGEs on keratinocytes [23]. Moreover, the constant paracrine signaling likely transduces stress signals from fibroblasts to keratinocytes [24].

In particular, the basement membrane appeared thicker in glycated RHS, although RHS in general lacks the sub-basal laminae of the basement membrane [25]. This alteration is well in accordance with the increased collagen-IV expression in glycated RHS [23], but the ultrastructural analysis identified longitudinal clusters of finest collagen fibrils to mimic a thick *lamina fibroreticularis* (fibroblast derived) of the basement membrane (Figure 3). Increased laminin-5 expression (Figure 4) nicely supports this hypothesis. As one of the principal structural elements of the basement membrane, laminin establishes networks with other proteins like collagens and determines the basement membranes common structure [26]. It is tempting to speculate that these structures emulate the age-specific reduplication of the lamina densa and its associated anchoring fibril complex as observed in vivo [27].

Moreover, we observed enhanced keratinocyte proliferation in glycated RHS, indicated by the increase in total epidermal thickness (Figure 2 and Figure 4). In our study, more than 20 cell layers corresponded to the *stratum granulosum*, containing disseminated electron dense material, which we interpreted as atypical keratohyalin granules. Absent keratohyalin and increased *stratum granulosum* height were described in particular types of psoriatic epidermis [28,29]. The increased thickness of the *stratum granulosum* indicates defective differentiation of the transitional located keratinocytes of the *stratum granulosum* and thus resembles parakeratotic psoriatic skin. The increased expression of loricrin was well in accordance with the abundant fine granula in the epidermal compartment of glycated RHS (Figure 5) [30]. In fact, keratinocytes with atypical keratohyalin granules dominated the epidermal compartment as “*stratum intermedium*” of glycated RHS. Furthermore, we observed changes in the intercellular space of the *stratum corneum* (Figure 4), which intrigued us to investigate the *stratum corneum* lipid order and profile.

We found an apparent, although not statistically significant, trend to increased Cer amounts in glycated RHS, being accompanied by the significantly improved lipid order (Figure 6). The apparent increase in Cer levels without a change in the SM and GCer concentrations suggests enhanced Cer synthesis de novo, with similar activities of sphingomyelinase and β-glucocerebrosidase. The improved chain order of barrier lipids would explain the trend to reduced permeability of glycated RHS as the membranes with fully stretched all-trans lipid chains would provide a stronger barrier to permeating substances than that with more fluid mobile lipids. This was applied to small molecules like caffeine, a recommended test compound for percutaneous absorption experiments by the Organisation for Economic Co-operation and Development, as well as to Bodipy 493/503, which shares the physicochemical properties of glucocorticoids. As with non-glycated RHS, macromolecules like CMS nanotransporters do not surmount the *stratum corneum* (Figure 7). This is well in accordance with previous models with intact skin barrier and suggests a penetration enhancing effect of CMS nanotransporters beyond simply carrying their cargo [31].

Our results are well in accordance with previous ribose-based approaches, but are not in line with glyoxal induced effects [6,32]. Moreover, we found a deviating *stratum corneum* lipid profile from glycated reconstructed human epidermis [7]. These changes might be attributed to the glycation method or the fact that reconstructed human epidermis lack the cross-talk between fibroblasts and keratinocytes. Even glyoxal impaired the skin barrier function in excised murine skin only after 72 h of direct exposure [7]. Culturing reconstructed human epidermis on a basement membrane surrogate, glycated by glyceraldehyde, resulted in decreased filaggrin expression and skin barrier function [10]. Although we found similar changes in the protein expressions, we could not confirm an impaired barrier function due to collagen glycation. In fact, neither caffeine (M_r_ 194; log*P* 0.08), Bodipy (M_r_ 262.1; log*P* 3.5), nor CMS nanotransporter (M_r_ 74,000) were absorbed better into glycated RHS compared with non-glycated RHS. As fluorescence readings provide only a first insight, further studies on the trans-epidermal resistance of glycated human skin ex vivo might explain these deviating results. Taking into account the cytotoxic effects of aldehydes on keratinocytes, the constructs in both aldehyde-based studies might overestimate the impaired barrier function due to glycation.

Glycation induced aged or diabetic phenotypes in the dermis, while the glycated dermal ECM did not cause these phenotypes in the epidermal compartment of RHS. The increased epidermal thickness with incomplete differentiation resembles a psoriasis-like epidermal morphology. Correlating to increased granulocyte-macrophage colony-stimulating factor and interleukin-6 expression, the reduced filaggrin expression hinted towards an inflammatory RHS phenotype [33,34]. Moreover, keratinocytes in the level of *stratum granulosum* also lacked normal keratohyalin granules and pointed to a disturbed keratinization process with defective tonofibrillar differentiation—similar to psoriatic skin [35]. Nevertheless, glycation alone would not be suitable to emulate psoriatic skin, because neither immune cells nor Th-17 cytokines were added to the culture. Moreover, glycated RHS did not show the impaired barrier function of psoriatic skin [36]. Nevertheless, a clinical study proved increased amounts of AGEs in patients with severe psoriasis [3], and AGEs appeared to fuel cutaneous inflammation [37].

In conclusion, our study adds several missing links to the effects of AGEs on the cutaneous morphology and skin barrier function. Based on our results, glycation of the ECM markedly affects epithelial differentiation, and thus should be considered when emulating (skin) diseases like diabetes, psoriasis, and skin cancer. The better understanding of the interactions between matrix and cells will help to improve the pharmacological treatment of these diseases. Finally, the ultrastructural analysis proved indispensable, especially for matrix effects, and should be used complementarily with molecular analyses in the characterization of human cell-based models.

## 4. Materials and Methods

### 4.1. Collagen Glycation

Collagen was glycated as described previously [9]. In brief, collagen G (4 mg bovine collagen per mL; Biochrom, Berlin, Germany) was incubated with 10 mM D-ribose (Sigma Aldrich, München, Germany) in phosphate-buffered saline (PBS) at room temperature for three weeks and protected against light. Moreover, collagen G was incubated with PBS only as negative control and with 10 mM glyoxal (Sigma Aldrich, München, Germany) for comparison. Subsequently, glycated collagen was extensively dialyzed in sterile water at 4 °C for 48 h, using Spectra/Por^®^ 4 Dry Standard RC Dialysis Tubing, 12–14 kD molecular weight cut-off, 32 mm flat width, 100 ft length (132703; Spectrum, Henderson, NV, USA). The sterile water was changed every 12 h. Following the dialysis, we subjected the samples to fluorescence analysis (Hitachi F-4500; Hitachi, Tokyo, Japan) to detect AGEs. Therefore, 1.5 mL glycated collagen was digested by pepsin (1 mg pepsin in 5 mL of 0.5 M acetic acid) at 37 °C overnight. The solution was centrifuged at 10,000× *g* for 5 min, the supernatant, containing the digested collagen, was measured at λ_ex_ 370 nm/λ _em_ 440 nm for total AGEs [9].

### 4.2. Cell Culture and Viability

Cell viability was assessed by 3-(4,5-dimethylthiazol-2-yl)-2,5 diphenyltetrazolium bromide (MTT; Sigma Aldrich, München, Germany) reduction as previously described [38]. In brief, NHK (passage 3) and NHDF (passage 3) were isolated from therapeutically indicated circumcision with permission (ethical approval EA1/081/13). Cell culture was performed according to standard operating procedures, and referring to good cell culture practice. We used 0.005% [*w*/*v*] sodium dodecyl sulphate (SDS) as positive control and 10% [*v*/*v*] double distilled, sterile water as solvent control.

### 4.3. Reconstructed Human Skin (RHS)

RHS was built in three steps [15]. First, 1 mL acellular collagen G or a 1:1 mixture of collagen G and glycated collagen G was poured into a cell-culture insert (Corning, Corning, NY, USA) and incubated for 2 h to form a gel. Second, fibroblasts (0.8 × 10^6^ cells per construct) were added to 3 mL collagen G or a 1:1 mixture of collagen G and glycated collagen G, poured onto the acellular collagen, and incubated for 24 h. The dermal equivalents were submerse cultured for seven days in construct growth medium (CGM: Dulbecco’s modified eagle’s medium /F12 + GlutaMax supplemented with 10% fetal calf serum, 1% Pen/Strep, adenine HCl monohydrate 40 µM, amphotericin B 30 µg/L, choleratoxin 0.1 nM, epidermal growth factor 10 µg/L, hydrocortisone 3.5 mg/L, insulin 4.4 mg/L, non-essential amino acids 0.5%, transferrin 4.4 mg/L, triiodothyronine 2 nM). Third, NHK (3.0 × 10^6^ per construct) were seeded onto the dermal compartment at day 8. RHS were raised to the air–liquid interface at day 10 and cultured in construct differentiation medium (CDM: CGM supplemented with ascorbic acid 0.25 mM, calcium chloride 2 mM). The culture medium was changed three times a week. At day 21, the constructs were subjected to further analyses as described below.

### 4.4. Reflectance Confocal Microscopy (RCM) and Immunolocalization of Proteins

RCM was performed with VivaScope^®^ 3000 (MAVIG; München, Germany). Subsequently, the constructs were snap frozen, sectioned into 7 μm slices (Leica CM 1510S; Wetzlar, Germany), and analyzed by immunofluorescence staining [15]. Antibodies against the following antigens were purchased from Abcam (Cambridge, UK): filaggrin (1:1000; ab81468), carboxymethyl lysine (1:100; ab27684), human integrin beta 1 (1:100; ab3167); and from Thermo Fisher Scientific (Darmstadt, Germany): loricrin (1:500; PA5-34945). Immunofluorescence staining was performed according to standard protocols. Pictures were taken with a fluorescence microscope (BZ-8000, Keyence; Keyence, Neu-Isenburg, Germany) and analyzed with BZAnalyzer and ImageJ software. Five individual measurements per construct were performed observer-blinded (RBR, CH, CW, CZ).

### 4.5. Transmission Electron Microscopy (TEM)

RHS was fixed in Karnovsky solution (7.5% glutaraldehyde and 3% paraformaldehyde in phosphate buffered saline) for 4 h, then washed in 0.1 M cacodylate buffer (cacodylic acid sodium salt trihydrate; Roth, Karlsruhe, Germany) and incubated in 1% osmium tetroxide (Chempur; Karlsruhe, Germany) for 120 min. Dehydration was performed in an ascending series of ethanol. Finally, the models were washed in the intermedium propylene oxide (1,2 Epoxypropan; VWR, Darmstadt, Germany).

Embedding of RHS was performed in a mixture of agar 100 (epoxy resin), DDSA (softener), Epoxy embedding medium (hardener), and DMP 30 (catalyst) (all: Agar Scientific, Stansted, UK), followed by polymerization at 45 °C and 55 °C, each for 24 h. Semi- and ultrathin sections were cut using an ultra-microtome Reichert Ultracut S (Leica, Wetzlar, Germany) and the semi-thin sections (0.5 µm) were stained with modified Richardson solution [39] for 45 s at 80 °C.

Semi-thin sections were examined under light microscopy Olympus CX21 (Olympus, Stuttgart, Germany). Next, ultrathin (80 nm) sections were mounted on nickel-grids (Agar Scientific). The RHS morphology was assessed with an electron microscope (ZeissEM109, Oberkochen, Germany). Images were taken and processed using Adobe^®^ Photoshop^®^ (Adobe^®^ Systems, San José, CA, USA).

### 4.6. Carboxymethyl Lysine Quantification

RHS was separated into epidermal and dermal compartments and dissected. Thereafter, constructs were transferred to tubes containing 300 µL (epidermis) or 700 µL (dermis) PBS and a 5 mm stainless steel bead and immediately disintegrated using Tissue Lyser II (Qiagen, Hilden, Germany) at 25 Hz for 5 min. The lysed suspensions were analyzed for *N*-carboxymethyl-lysine content by OxiSelect™ ELISA (Cell Biolabs; San Diego, CA, USA) according to the manufacturers’ instructions.

### 4.7. Stratum Corneum Lipid Analysis

**Figure 8:**
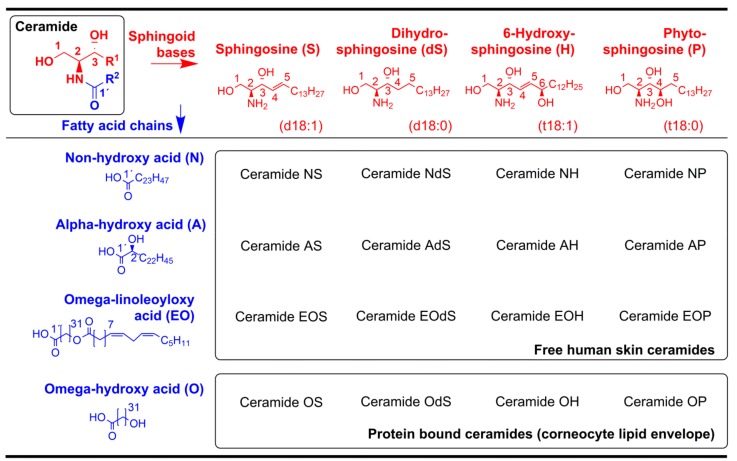
Ceramide nomenclature. Reprinted from the literature [15], Copyright 2016, with permission from Elsevier.

The *stratum corneum* of RHS was isolated, and subjected to infrared spectroscopy using a Nicolet 6700 spectrometer (Thermo Fisher Scientific, Waltham, MA, USA) equipped with a single-reflection MIRacle attenuated total reflectance ZnSe crystal (PIKE technologies, Madison, WI, USA) at 23 °C. The spectra were generated by co-addition of 256 scans collected at 4 cm^-1^ resolution and analyzed with the Bruker OPUS software. The exact peak positions were determined from second derivative spectra. The lipids were then extracted and analyzed by high performance thin layer chromatography (HPTLC; [40]). Briefly, the analysis was performed on silica gel 60 HPTLC plates (20 × 10 cm, Merck, Darmstadt, Germany). Cer subclasses, FFA, and Chol were separated using CHCl_3_/MeOH/acetic acid 190:9:1.5 (*v*/*v*/*v*) mobile phase twice to the top of the plate, whereas GCer, SM, PL, and CholS were separated using CHCl_3_/MeOH/acetic acid/H_2_O 65:25:6:3. The lipids were visualized by dipping in a derivatization reagent (7.5% CuSO_4_, 8% H_3_PO_4_, 10% MeOH in water) for 10 s, heating at 160 °C for 30 min, and quantitated by densitometry using TLC scanner 3 and WinCats software (Camag, Muttenz, Switzerland). For the used standard lipids and their calibration curve ranges, see Table 1; for ceramide nomenclature, see Figure 8. Cer and GCer were purchased from Avanti Polar Lipids (Alabaster, AL, USA). All other chemicals, including lignoceric acid, Chol, CholS, SM, and PL, were purchased from Sigma Aldrich (München, Germany). Non-commercially available Cer EOS and NH standards were synthesized according to previously published methods [41,42].

### 4.8. Skin Permeation

The permeation of radiolabeled [1-methyl^14^C]caffeine (*M*_r_ 194; log*P* 0.08; PerkinElmer, Waltham, MA, USA) was studied according to the validation study on the use of skin models for skin absorption testing [39]. In brief, tissue integrity of RHS was evaluated prior to mounting RHS in static Franz cells (∅ = 15 mm, with receptor chamber volume = 12 cm^3^; PermeGear, Bethlehem, PA, USA). The stratum corneum was placed facing the air and the dermal compartment of RHS was in contact with the supporting membrane and the receptor medium PBS. The receptor medium was kept at a constant temperature of 33.5 ± 0.5 °C using a temperature-controlled water jacket, and stirred with a magnetic bar at 500 rpm. The absence of air bubbles was monitored throughout the experiment. Then, 281.4 μg/cm^2^ caffeine was applied in aqueous solution onto the RHS surface. The opening of the Franz cell was covered by Parafilm^®^ and the caffeine permeation was quantified from the receptor medium by radiochemical detection (Hidex 300 SL liquid scintillation counter; HIDEX, Turku, Finland).

### 4.9. Penetration of Bodipy Loaded Dendritic Core-Multishell Nanotransporters (Bodipy-CMS)

Bodipy 493/503 (λ_ex_ 493 nm; λ_em_ 503, M_r_ 262.1, log*P* 3.5, Thermo Fisher Scientific; Darmstadt, Germany) was dispersed in base cream (13.6 mg/L Bodipy; 40.0% purified water, 25.5% white vaseline; 10.0% propylenglycol; 7.5% medium chain triglycerides; 7.0% macrogol-1000-glycerol monostearate; 6.0% cetyl alcohol; and 4.0% glycerol monostearate 60). Additionally, CMS nanotransporters [43] were labelled with indocarbocyanine and loaded with Bodipy (13.6 mg/L). Then, 30 μL/cm^2^ of test formulations was applied onto the RHS surface and remained there for 6 h according to a previously published protocol [31]. Pictures were taken with a fluorescence microscope (BZ-8000, Keyence; Keyence, Neu-Isenburg, Germany) and analyzed with ImageJ software. Five individual measurements per construct were performed observer-blinded (RBR, CW, CZ).

### 4.10. Statistical Analysis

Data are presented as the mean ± SD from two to four independent experiments. The Mann–Whitney test or Kruskal–Wallis test with Dunn’s Post hoc test was used to indicate statistically significant differences (*p* ≤ 0.05).

## Figures and Tables

**Table 1:** Calibration curve ranges of lipid standards used for high performance thin layer chromatography (HPTLC) analysis. FFA—free fatty acid.

Lipid Standard		Calibration Curve Range [µg]
FFA	Lignoceric acid	0.5–12.5
Chol	Cholesterol	0.5–12.5
Cer	Ceramide EOS	0.05–1.25
	Ceramide NS	0.2–5
	Ceramide NP	0.4–9.5
	Ceramide AS	0.1–2.5
	Ceramide NH	0.15–4
	Ceramide AP	0.1–2.5
CholS	Cholesterol sulphate	0.105–2.474
SM	Sphingomyelin	0.211–4.947
GCer	Glucosylceramide	0.263–6.184
PL	Phospholipid	0.421–9.895

## Abbreviations

AGEs	Advanced Glycated End-products
CML	N-CarboxyMethyl-Lysine
CMS	Dendritic Core-Multishell
DAPI	4′,6-DiAmidino-2-PhenylIndole
ECM	ExtraCellular Matrix
HPTLC	High-Performance Thin-Layer Chromatography
NHDF	Normal Human Dermal Fibroblast
NHK	Normal Human Keratinocytes
PBS	Phosphate-Buffered Saline
RAGE	Receptor of AGEs
RHS	Reconstructed Human Skin
SDS	Sodium Dodecyl Sulphate
TEM	Transmission Electron Microscopy
